# Myeloid-derived suppressor cells: Cancer, autoimmune diseases, and more

**DOI:** 10.18632/oncotarget.28303

**Published:** 2022-11-17

**Authors:** Masahiko Shibata, Kotaro Nanno, Daigo Yoshimori, Takahiro Nakajima, Makoto Takada, Takashi Yazawa, Kousaku Mimura, Norio Inoue, Takafumi Watanabe, Kazunoshin Tachibana, Satoshi Muto, Tomoyuki Momma, Yoshiyuki Suzuki, Koji Kono, Shungo Endo, Seiichi Takenoshita

**Affiliations:** ^1^Department of Comprehensive Cancer Treatment and Research at Aizu, Fukushima Medical University, Fukushima, Japan; ^2^Department of Surgery, Cancer Treatment Center, Aizu Chuo Hospital, Fukushima, Japan; ^3^Department of Gastrointestinal Tract Surgery, Fukushima Medical University, Fukushima, Japan; ^4^Aizu Oncology Consortium, Fukushima, Japan; ^5^Department of Surgery, Nippon Medical School, Tokyo, Japan; ^6^Department of Surgery, Bange Kousei General Hospital, Fukushima, Japan; ^7^Department of Obstetrics and Gynecology, Fukushima Medical University, Fukushima, Japan; ^8^Department of Breast Surgery, Fukushima Medical University, Fukushima, Japan; ^9^Department of Chest Surgery, Fukushima Medical University, Fukushima, Japan; ^10^Department of Radiation Oncology, Fukushima Medical University, Fukushima, Japan; ^11^Department of Colorectoanal Surgery, Aizu Medical Center, Fukushima Medical University, Fukushima, Japan; ^12^Fukushima Medical University, Fukushima, Japan

**Keywords:** MDSC, immunosuppression, Treg, TAM, cancer

## Abstract

Although cancer immunotherapy using immune checkpoint inhibitors (ICIs) has been recognized as one of the major treatment modalities for malignant diseases, the clinical outcome is not uniform in all cancer patients. Myeloid-derived suppressor cells (MDSCs) represent a heterogeneous population of immature myeloid cells that possess various strong immunosuppressive activities involving multiple immunocompetent cells that are significantly accumulated in patients who did not respond well to cancer immunotherapies. We reviewed the perspective of MDSCs with emerging evidence in this review.

Many studies on MDSCs were performed in malignant diseases. Substantial studies on the participation of MDSCs on non-malignant diseases such as chronic infection and autoimmune diseases, and physiological roles in obesity, aging, pregnancy and neonates have yet to be reported. With the growing understanding of the roles of MDSCs, variable therapeutic strategies and agents targeting MDSCs are being investigated, some of which have been used in clinical trials. More studies are required in order to develop more effective strategies against MDSCs.

## INTRODUCTION

Cancer immunotherapy is one of the major treatment modalities for malignant diseases [1–3]; however, its clinical outcome is not uniformal in all cancer patients. Myeloid-derived suppressor cells (MDSCs) represent a heterogeneous population of immature myeloid cells that possess various strong immunosuppressive activities involving multiple immunocompetent cells and are significantly accumulated in patients who did not respond well to cancer immunotherapies [4, 5]. Although many studies have been conducted on MDSCs in malignant diseases, substantial studies on non-malignant conditions such as chronic infection, sepsis and autoimmune diseases, and the physiological roles in obesity, aging, pregnancy and neonates have reported on the participation of MDSC [6].

The tumor microenvironment (TME) is the environment among tumor cells, including blood and lymphatic vessels, soluble mediators such as cytokines and chemokines, immune competent cells such as dendritic cells, B lymphocytes and T lymphocytes, and immune suppressor cells including regulatory T-cells (Tregs), macrophages and MDSCs. MDSCs migrate to the TME, activated and proliferated by the soluble mediators, and orchestrate a wide variety of immune cells towards immunosuppression in the TME [7]. Here, we review the following: the phenotypes and origins of MDSCs; the mechanisms of immunosuppression by MDSCs; MDSC functions in the TME; MDSCs in benign disorders and physiology; and consideration of MDSC manipulation in cancer treatment.

## PHENOTYPES AND ORIGINS OF MDSCS

Myeloid cells are heterogeneous and multipotent population. Mononuclear myeloid cells differentiate into monocytes, macrophages and dendritic cells (DC) under various inflammatory conditions. Granulocytic myeloid cells are cells that differentiate into polymorphonuclear neutrophils, basophils, eosinophils and mast cells.

A significantly increased amount of host immunocompetent cells combined with the advancement of malignant diseases have been reported since 1929. Sonnenfeld et al., first reported leukemoid reactions [8] and Robinson et al., reported granulocytosis [9]. Later on, these abnormal myeloid cells were named natural suppressor (NS) cells, which suppress the proliferation and activation of lymphocytes, as well as the induction of cytotoxic T lymphocytes (CTL) [10].

Involvement of myeloid cells on the immunosuppression of cancer patients has been reported since the 1970s [11]. The phenotypic characterization of NS cells was initially performed in mice and afterwards NS cells were recognized as committed myeloid progenitor cells [12]. In tumor-bearing mice, these cells have been found to increase along with tumor progression and expand with hematopoietic growth factors such as granulocyte-macrophage colony-stimulating factor (GM-CSF) and granulocyte-CSF (G-CSF) [13, 14]. Cells that induce T-cell dysfunction have been reported to cover immature myeloid cells (iMCs), myeloid suppressor cells (MSCs), and Gr1+ myeloid cells [15, 16]. In 1998, Bronte et al., suggested that the phenotype of the population of iMCs is Gr1+CD11b+ [17], and Gabrilovich et al., reported on MSCs in 2007 [18]. Then the terminology for myeloid-derived suppressor cells, and a consensus [19] was made by others in same journal issue of 2007, and the controversy in nomenclature reached identifying MDSCs as the term of these cells. MDSCs have been described as heterogeneous [11], and these cells are divided into monocytic MDSCs (M-MDSCs) that have a monocyte-like morphology and express CD11b+Ly-6G^low^Ly-6C^high^, and granulocytic MDSCs (G-MDSCs) that have a granulocyte-like morphology and express CD11b+Ly6G+Ly6C^low^. The phenotypic characterizations of these two populations are detailed in Table 1. Both populations possess immunosuppressive activity of T-cells. M-MDSCs express nitric oxide synthase 2 (NOS2) and G-MDSCs express arginase type 1 (ARG1) [20] (Table 2). In most tumor-bearing mouse models. G-MDSCs are significantly increased, and thus the G-MDSC subset is generally accepted as the predominant MDSC population in a cancer-bearing host. Gr-1 staining is also useful for identifying these two fractions of MDSCs in mice; the Gr-1^Br^ subset is mainly composed of G-MDSCs and the Gr-1^int^ subset is mainly composed of M-MDSCs [21]. Furthermore, subset markers of F4/80 and MHC-II are used to distinguish between M-MDSCs and tumor-associated macrophages (TAMs) [22].

**Table 1:** Phenotypic characteristics of monocytic (M-) and granulocytic (G-) myeloid-derived suppressor cells (MDSC)

	Murine		Human	
	M-MDSC	G-MDSC	M-MDSC	G-MDSC
CCR2	+++	+++	++	–
CXCR2	+++	+++		
CXCR4	+++	+++	+++	+++
CD11b	+++	+++	+++	+++
CD14			+++	Low
CD15			–	+++
CD16			Low	Low
CD33			+++	+++
CD39			+++	+++
CD45	+++	+++	+++	+++
CD62	Low	Low	Low	Low
CD80	+++	+++		
CD115(M-CSFR1)	++	–	++	–
CD117	–	–	Low	++
CD124(IL-4R)	+++	+++	+++	+++
Ly6C	+++	Low		
Ly6G	Low	+++		
MHC I	+++	+++		
HLA-DR			–	–
Tie-2	–	–	+++	–
VEGFR1	+++	+++	+++	+++
VEGFR2	+++	+++	+++	+++
Gr1	Int	Br	–	–

The table shows phenotypic markers of murine and human MDSC (monocytic and granulocytic). These phenotypic markers have been used to identify MDSCs. The expressions of markers sometimes have been expressed as low: Low, intermediate: Int or bright: Br. Abbreviations: M-CSFR1: macrophage-colony stimulating factor receptor 1; VEGFR: vascular endothelial growth factor receptor.

**Table 2:** Functional differences between M-MDSC and G-MDSC

	M-MDSC	G-MDSC
Inducers	M-CSF, IL-6	G-CSF, IL-6
Inhibition of T cell proliferation	++	+
ROS	–	++
MPO	–	++
ARG1	+	++
NOS2	++	–
Immune cell polarization	TAM, DC	TAN, PMN

The table shows the functional differences between M-MDSC and G-MDSC. The inhibitory action of T cell proliferation is stronger in M-MDSC and the differences on the expressions of ROS and MPO are shown. M-MDSC is induced by M-CSF and IL-6 whereas G-MDSC is by G-CSF and IL-6. M-MDSC and G-MDSC polarize into TAM and DC, and TAN and PWN, respectively. Abbreviations: MDSC: monocyte-derived suppressor cells; M-MDSC: monocytic MDSC; G-MDSC, granulocytic MDSC; M-CSF: macrophage colony stimulating factor; G-CSF: granulocyte colony stimulating factor; TAM: tumor associated macrophage; DC: dendritic cells; TAN: tumor associated neutrophil; PMN: polymorphonuclear neutrophil; ROS: reactive oxygen species; MPO: myeloperoxidase; ARG1: Arginase type 1; NOS2: nitric oxide synthetase 2.

In human cancer, identification of MDSCs by CD34 expression was attempted [23], but this marker was not used much since the CD34+ subset may include hematopoietic progenitor cells. Thereafter. the expression of human leukocyte antigen D-related (HLA-DR) and myeloid marker CD33 were used to identify MDSCs in human (Table 1) [24]. In this fashion, human MDSCs were identified as HLA-DR-CD33+ or CD14-CD11b+ cells [25]. Moreover, new markers have been identified for MDSCs in human cancers; CD14^dull^ for M-MDSC and CD15+ for G-MDSCs [26].

Rapid terminal differentiation of myeloid cells typically physiologically appears after acute infection, stress, or trauma. On the other hand, pathological myelopoiesis with defective myeloid cell differentiation typically appear during chronic inflammation or cancer, and is characterized by infiltration of MDSCs in tumor tissue or on sites of chronic inflammation [27]. This abnormal myelopoiesis in cancer and chronic inflammation is induced by several cytokines, including interleukin (IL)-17A, G-CSF, GM-CSF, and TNF (tumor necrosis factor)-α, as well as transcription factor ROR1C [28].

Condamine et al., reported the “two-signal model” for the mechanism of MDSC accumulation in 2015 [29]. This model demonstrated two distinct types of signals, physiological response to the increased immature myeloid cells due to the inhibition of terminal differentiation, and pathological activation of myeloid cells to be converted to MDSCs. It also reported that the first signals are mostly mediated by tumor-derived growth factors and that the second signals are driven by the soluble factors produced by the tumor stroma [5, 30]. Furthermore, plasticity of MDSCs is recently discussed and the hypothesis that MDSCs could also be derived from mature myeloid cells such as monocytes and neutrophils with certain signals, not from immature myeloid cells including hematopoietic stem cells and progenitor cells, was reported in 2017 and 2018 [31, 32].

## MECHANISMS OF IMMUNOSUPPRESSION BY MDSCS

Both M-MDSC and G-MDSC have been shown to possess multiple suppressing activities for T lymphocytes. The suppressing function of MDSCs has now been summarized in the following four actions. The first action is production of ARG1, that induces the depletion of L-arginine. Subsequently, the downregulation of CD247, a subunit of natural killer receptors NKp46, NKp30 and TcγIII of NK cells and T-cells, will be made and result in the inhibition of the proliferation of NK cells and T-cells [32]. The second action is production of reactive oxygen species (ROS) and nitric oxide (NO) that drive the nitration of the FcγIIIA-associated molecule, leading to the deactivation of T-cells and NK cells [32]. Moreover, NO produced by MDSCs nitrate STAT (signal transducers and activators of transcription) 1, resulting in the decreased response to interferon (IFN)-γ of T-cells and NK cells. The third action is expansion of regulatory T (T reg) cells and T helper (Th) 17 cells [33, 34] that induce multiple deactivation steps of CD8+T-cells. Finally, the fourth action is upregulated expression of PD-L1 on MDSCs that results in the inactivation and proliferation of T-cells [35]. The proportion of G-MDSC is much higher than that of M-MDSC in the peripheral lymphoid organs. G-MDSCs have moderate suppressing activity and suppression in tumor specific immune reactions is mostly driven by G-MDSC. In TME, the proportion of M-MDSCs is higher than that of G-MDSC and M-MDSCs are more suppressive than G-MDSCs [36].

Functional interactions between MDSCs and Tregs have mainly been explained by the actions of variable soluble factors such as transforming growth factor (TGF)-β and IL-10 [34]. IL-10 and TGF-β produced by MDSCs promote induction, proliferation and activation of Tregs. The production of IL-10 and TGF-β enhance the generation of MDSCs. These cytokines increase the expression of PD-L1 on MDSCs. In addition to these soluble mediators, interactions of MDSCs and Tregs are also performed by metabolic crosstalk, described in the next section, and direct cell-to-cell contact. In the pancreatic ductal adenocarcinoma (PDAC) model, physical interactions between MDSCs and Treg cells were demonstrated in multiple ways including videomicroscopic analysis [37]. It was concluded that cell-to-cell contact is necessary for MDSCs-mediated induction of Tregs. Furthermore, interactions between MDSCs and Th17 cells have also been studied and discussed. Th17 cells, another subset of T helper cells, are characterized by production of IL-17 and its transcription factor RORγt [38]. We have reported that the production of IL-17 by peripheral blood mononuclear cells (PBMC) is significantly increased in patients with various types of cancer compared to healthy volunteers. Moreover, the levels of IL-17 were significantly correlated with the numbers of circulating MDSCs in these patients and therefore, it is speculated that a functional connection of Th17 cells and MDSCs may exist [38, 39]. Wen et al., reported the interplay between MDSCs and Th17 cells in several pathological conditions, including tumors [40] and that the increased infiltration of MDSCs in TME is usually accompanied with an increased accumulation of Th17 cells. However, this association has not been confirmed, and remains controversial.

## MDSCS FUNCTIONS IN TME

TME is a complex environment composed of tumor cells, cancer-associated fibroblasts (CAF), endothelial cells and immunocompetent cells that form tumor stroma. The stroma is predominantly characterized by hypoxia, acidity, IDO (indoleamine 2,3-dioxygenase)-dominant condition and low levels of tryptophan and glucose [41]. These important characteristics are symphonized to be immunosuppressive involving immunosuppressive cells such as MDSCs, Tregs and tumor associated macrophage (TAM)s. These cells exhibit crosstalk with each other and regulate the function of individual cells located in the TME. The TME is predominantly characterized by accumulation of adenosine. Adenosine converted from ATP is released from apoptotic cancer cells and subsequently degraded through binding with CD39 and CD73 in TME. High production of adenosine in the TME is immunosuppressive since it is harmful to CD8+ T cells and induces the activation and proliferation of Tregs. Since both MDSCs and Tregs express CD39 and CD73, it has been reported that an accumulation of adenosine induces the infiltration of MDSCs and Tregs in the TME and increases their immunosuppressive activities [42, 43]. Moreover, TGF-β produced by tumor cells and MDSCs increase the expression of CD 39 and CD73 on MDSC, promoting further accumulation of adenosine in TME. Thus, the production of adenosine serves an additional mechanism of immunosuppression mediated by MDSCs. In addition to the metabolic interaction of MDSCs and Tregs, interactions between these two fractions regulated by direct contact have also been reported. It has been suggested that the presence of Tregs increases the acquisition of more immunosuppressive MDSCs with elevated expression of PD-L1 [43]. On the other hand, MDSCs have been reported to enhance the immunosuppressive properties of Tregs through the direct interaction of CTLA (cytotoxic T-lymphocyte-associated protein)-4 and CD80 on MDSCs [44]. The positive interactions of MDSCs and Tregs have further been proven by studies using other immune checkpoint molecules including VISTA (V-domain Ig suppressor of T-cell activation), TIM (T-cell immunoglobulin and mucin domain)-3, TIGIT (T-cell immunoreceptor with immunoglobulin and ITIM domains) and LAG-3 (lymphocyte-activation gene-3) as negative regulators of T-cell function [45]. In the TME, the generation of MDSCs from myeloid precursors is stimulated by various soluble factors produced by tumor cells such as cytokines and growth factors. These factors include TNF-α, macrophage colony-stimulating factor (M-CSF), GM-CSF, VEGF (vascular endothelial growth factor), prostaglandin E2 (PGE2), and interleukins (IL-1β, IL-10, IL-18, and IL-6)[30]. The migration of MDSCs into the TME have been reported to be driven by chemokines such as CXC-ligand (CXCL1, CXCL8, CXCL12), CC-ligand (CCL1, CCL2, CCL3, CCL5, CCL7), and their receptors, CCR2, CCR5 and CXCR4 [30].

The structure and functions of blood vessels in the TME are very different from those in non-cancerous tissue, and the abnormal vasculature in the TME both promotes tumor progression and produces distant metastasis in many ways [46]. The decreased perfusion is seen in abnormal vasculature in the TME, and leads to hypoxia and acidosis. These are important characteristics for local immunosuppression involving the impairment of CT8+T-cells, as well as the activation and expansion of MDSCs, Tregs and TAMs. Various types of cytokines and chemokines are also produced in the TME by this pathological environment. VEGF, a well-studied member among these soluble factors that is produced by various sources, such as tumor cells and Tregs, induces further hypoxia and acidosis in the TME. It has been reported that receptor molecules for VEGF (VEGF-R1 or VEGF-R2) are expressed in MDSCs, Tregs and TAMs, and that VEGF promotes the induction, expansion and activation of MDSC and immunosuppression in the TME [46–48].

## MDSCS IN BENIGN DISORDERS AND PHYSIOLOGY

MDSCs have been reported to be increased in patients with several types of autoimmune diseases such as type 1 diabetes, rheumatoid arthritis (RA), systemic lupus erythematosus (SLE), multiple sclerosis (MS), inflammatory bowel disease (IBD) and autoimmune hepatitis [7]. Frequencies of MDSCs are elevated in patients with RA and SLE, and are associated with disease advancement [49, 50]. Th17 cells, originally reported as a cause of some autoimmune diseases, have also been examined in RA and the numbers of Th17 were reported to be inversely correlated to the frequencies of MDSCs [51]. It has also been reported that MDSCs have a proinflammatory role and may promote arthritis with the support of Th17 cells [49].

In MS patients, frequencies of MDSCs in relapsing patients were significantly higher than those in stable MS patients [52]. In a murine model of asthma, increased frequencies of G-MDSCs are closely correlated with elevation of Th17 cells [53]. Furthermore, it was shown that there was a significant accumulation of MDSCs in SLE, asthma, experimental autoimmune encephalitis (EAE), and collagen-induced arthritis in mouse models, where the accumulation of Th17 was significantly correlated to MDSCs frequencies, as well as disease severity [53–56]. MDSCs have also been highlighted in the onset or progression of IBD. In experimental colonic inflammation, the reduction of severity by anti-inflammatory agents was found to be associated with the accumulation of MDSCs [57, 58]. The experimental challenges of the adoptive transfer of MDSCs were made in the models of arthritis and inflammation of the lungs and liver, and revealed a significant reduction of inflammation [59, 60].

Since MDSCs are implicated in the onset and progress of several autoimmune diseases, as mentioned above, trials utilizing MDSCs may be important for patients who did not respond well to conventional treatments with drugs. Recently, it was reported that group 2 innate lymphoid cells (ILC2s) play a major role in asthma induction [61]. G-MDSCs were proven to inhibit the production of inflammatory cytokines by ILC2s and the adoptive transfer of MDSCs reduced the severity of allergic inflammation in the mouse models [62].

Interesting results, according to a complex interplay amongst metabolism and immune responses, have been reported. The pathological metabolism of tumor cells and immunocompetent cells in the TME has been described as enhancing the orchestration of tumor cell growth, pathological angiogenesis, chronic inflammation, and immune dysfunction [63–65].

**Figure 1:**
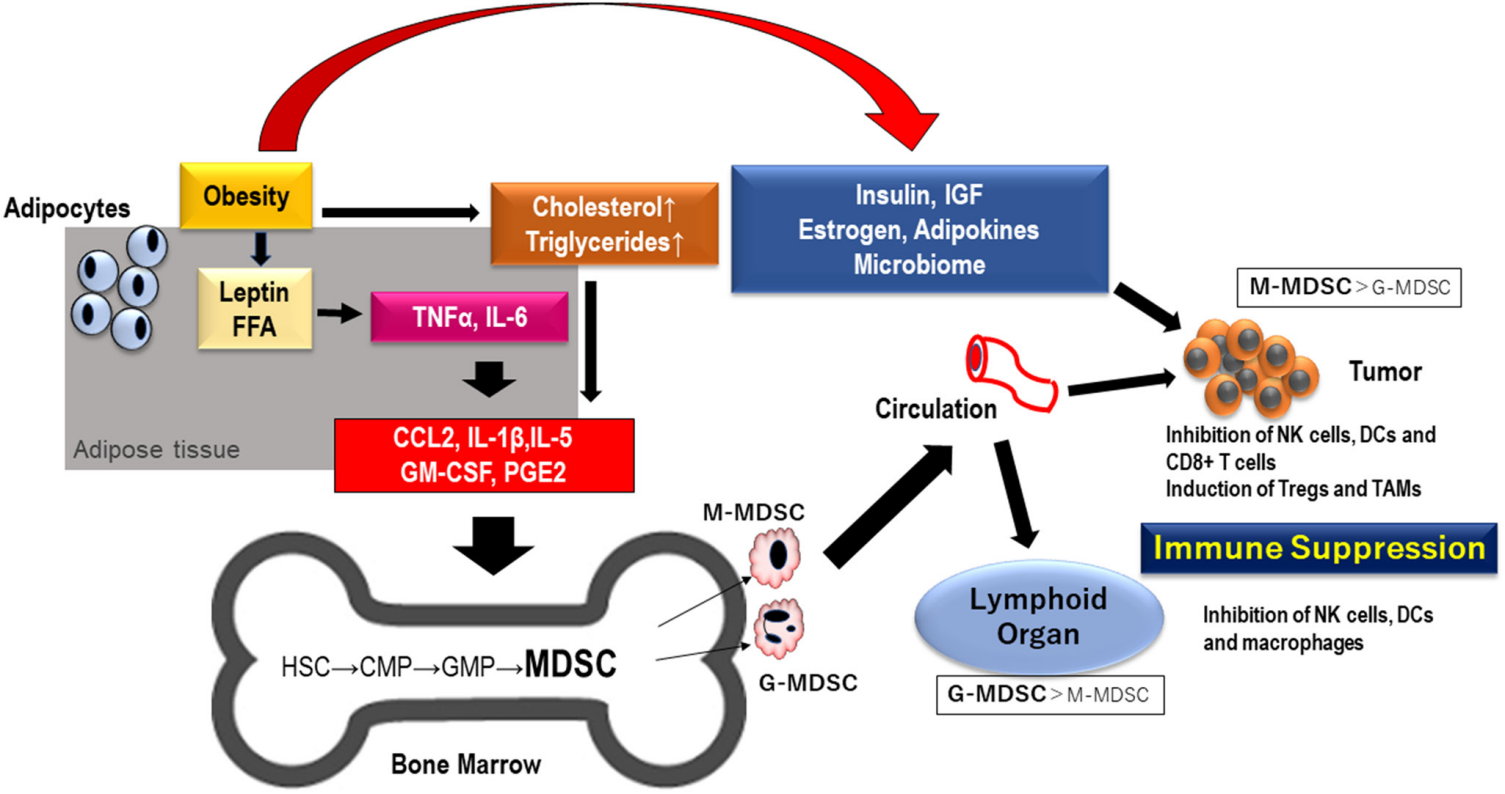
MDSCs generated by obesity migrate to lymphoid organ and TME. Inflammation driven by obesity cause the differentiation of MDSC in bone marrow through proinflammatory mediators including cytokines and chemokines. MDSC migrate into tumor tissue and lymphoid organ and result in immunosuppression in TME and lymphnode. Abbreviations: FFA: free fatty acid; TNF: tumor necrosis factor; IGF: insulin-like growth factor; IL: interleukin; CCL2: CC chemokine ligand 2; GM-CSF: granulocyte-macrophage colony stimulating factor; PGE: prostaglandin E; HSC: hematopoietic stem cell; CMP: common myeloid progenitor; GMP: granulocyte-monocyte progenitor; MDSC: myeloid-derived suppressor cells; NK: natural killer; Tregs: regulatory T cells; TAM: tumor associated macrophages; TME: tumor microenvironment.

Obesity, a pathological condition of uncontrolled lipid storage in adipose tissue, is described as having a strong association with insulin resistance and high blood levels of triglycerides and cholesterol [66]. It has been reported that the survival of obese cancer patients is worse than that of those without obesity, and that chronic inflammation and oxidative stress are important factors in tumor progression, especially in obese patients [65–67]. Obesity induces chronic inflammation through a network of cytokines, chemokines, and adipokines that stimulate the immunosuppression by MDSCs. Free fatty acids and leptin derived from adipocytes stimulate macrophages in adipose tissue to produce TNF-α and IL-6, and monocytes, macrophages and T-cells are accumulated into adipose tissues [66]. These immunocompetent cells (monocytes, macrophages and T-cells in adipose tissue) accumulated in adipose tissues promote chronic inflammation through further production of TNF-α and IL-6, and these cells also produce CC chemokine ligand 2 (CCL2), IL-1β, IL-5, GM-CSF and prostaglandin E2 that induce differentiation of MDSC from hematopoietic stem cells (HSC) and progenitor cells (CMP, common myeloid progenitor cells and GMP, granulocyte-monocyte progenitor cells) in bone marrow. Some of these soluble mediators also promote tumor progression (Figure 1) [68–70]. TAM and MDSCs also activate fatty acid oxidation with several molecules towards immunosuppression [71].

A number of studies on MDSCs during pregnancy have been performed, and reported that MDSCs are critical in the function of materno-fetal tolerance by impairing T-cell responses [7, 72–75]. The numbers of MDSCs were demonstrated to be lower in spontaneously aborting mice than control mice and it was suggested that MDSCs play an important role in protecting the fetus during gestation [76]. It has thus been reported that MDSCs are necessary to provide materno-fetal tolerance to pregnant women; therefore, the expansion and activation of MDSCs may be effective for women with abnormal pregnancies and/or habitual abortion [6]. Trophoblast or progesterone was demonstrated to induce MDSC expansion through CXCR4 and its ligand CXCL12 [77]. G-CSF was reported to restore MDSC-levels in the models in which MDSCs were suppressed experimentally [72]. The role of STAT3 was reported to be to regulate MDSCs, and it was demonstrated that estradiol and progesterone are involved in the expansion of MDSCs through STAT3 [6, 75].

Surprisingly, MDSCs have been reported to be implicated in patients with coronavirus disease 2019 (COVID-19) and it was shown that G-MDSCs were increased in the blood and lungs of such patients [78, 79].

Recently, targeting lipid metabolism in MDSCs has been attempted in order to improve cancer immunotherapy, since metabolic reprogramming enhances the development of immune cells. Fatty acid transport protein 2 (FATP2), a long chain fatty acid transporter, is upregulated in MDSCs in the TME, and has been reported to regulate the immunosuppressive activities of MDSC [80]. An uptake of fatty acids is increased via FATP and PGE2 produced by MDSCs and the FATP2 inhibitor showed a reduction of tumor progression with and without a combination of ICIs in mouse models. The mechanisms of these results are reported to be a decreased production of PGE2 by MDSCs and an increased infiltration of CD8+ T cells in the TME [81].

Biguanides, commonly used for the treatment of type II diabetes, showed a decreased infiltration of MDSCs in the TME, and reduced the immunosuppressive action of MDSCs with a combination of immune checkpoint inhibitor (ICI) through several mechanisms [82–86].

## CONSIDERATION IN CANCER TREATMENT

Close correlations of MDSC accumulation and clinical outcome have been reported in patients with various types of cancer [87–89]. It has been demonstrated that MDSCs are correlated with the outcome of not only cancer immunotherapies with ICIs, but also those of chemotherapies including sunitinib, cisplatin, doxorubicin and others [90–95].

**Figure 2:**
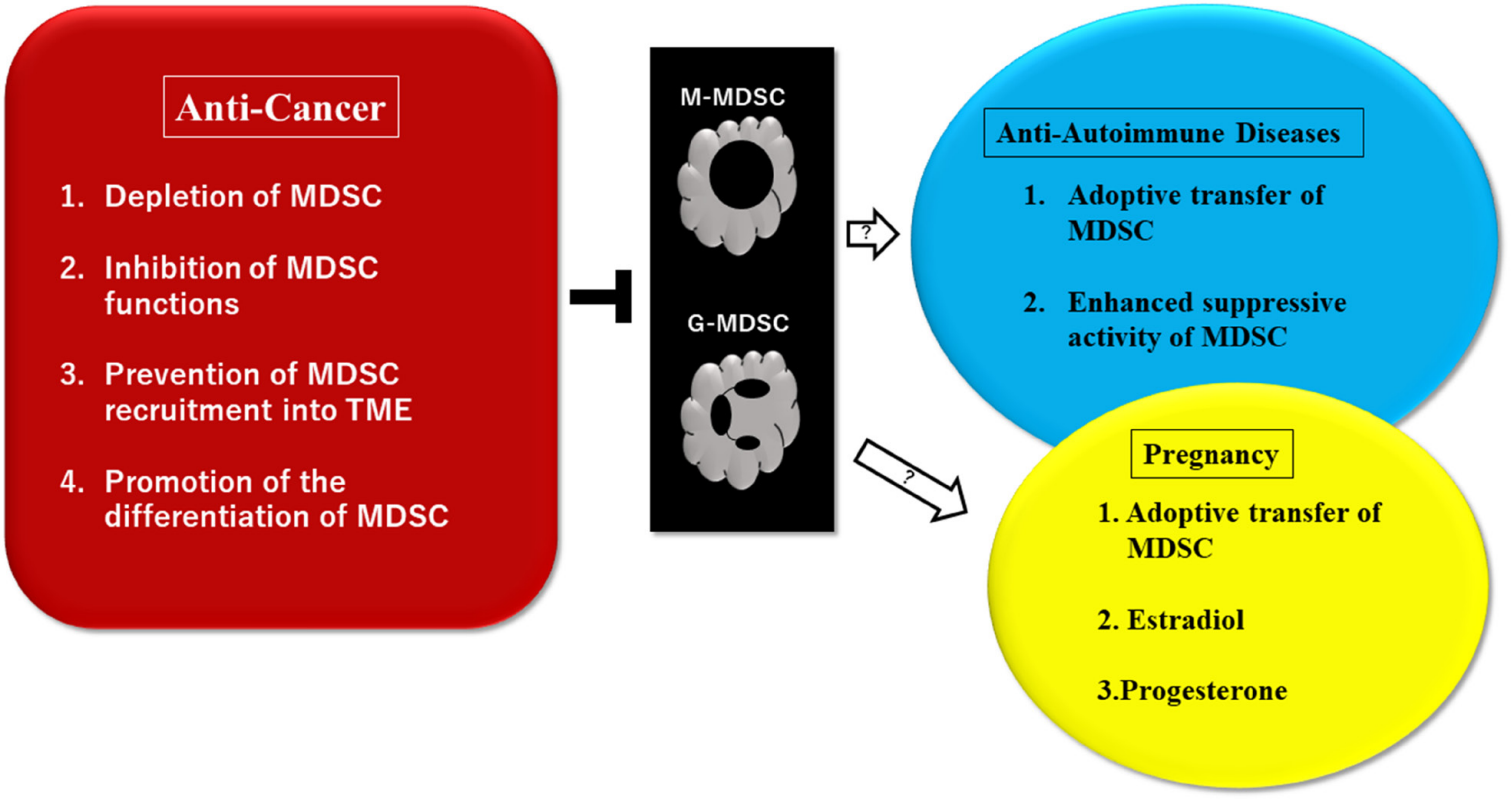
Potential therapeutic approaches to control myeloid-derived suppressor cells (MDSC)-driven pathological conditions of cancer, autoimmune diseases, and pregnancy. Multiple treatment approaches have been investigated in preclinical settings and clinical trials. Suppression of MDSC activities is a goal in the field of cancer therapy. Trials using MDSCs may be effective for patients where conventional treatments with drugs were not effective. MDSCs are necessary to provide materno-fetal tolerance to pregnant women and therefore, expansion and activation of MDSCs may be effective for women with abnormal pregnancies. It was reported that estradiol and progesterone are involved in the expansion of MDSCs through STAT3. Some ideas of treatments using MDSCs for pregnancies and autoimmune diseases are still in controversial. Abbreviations: MDSC: myeloid-derived suppressor cells (monocytic, granulocytic); TME: tumor microenvironment; STAT3: signal transducer and activator of transcription 3.

Suppression of MDSC activities is now a goal in the field of cancer therapy. De Cicco et al., reported that the approaches targeting MDSCs include 1. Depletion of MDSCs, 2. Inhibition of MDSC recruitment, 3. Inhibition of MDSC suppressing activity, 4. Promotion of differentiation of MDSC [96]. Veglia F, et al., reported that the effective targeting of MDSCs includes blocking the differentiation to MDSCs in the bone marrow, and inhibiting migration to the TME (Figure 2) [7].

The migration of G-MDSCs is mainly done by chemokine CXCL1, CXCL2 and IL-8 and the inhibition of CXCR2 is reported to improve the outcome of the effect of anti-PD-1 treatment against cancer [97–99]. Chemotherapeutic agents including 5-fluorouracil, carboplatin, paclitaxel, or gemcitabine have been demonstrated the ability to reduce circulating MDSC numbers and increase antitumor immune reaction [100–102]. CD33 is a human myeloid marker, and gamtuzumab (anti-CD33 antibody) conjugated with ozogamicin as shown to decrease MDSC in a phase 2 trial [103, 104].

All-trans retinoic acid (ATRA) that promotes the differentiation of MDSCs to macrophages and DCs showed decreased levels of MDSC in mice and humans [105, 106]. In cancer patients treated with tadalafil, PDE (phosphodiesterase)-5 inhibitor, the circulating levels of MDSCs, iNOS levels, and the expression of arginine on MDSCs were decreased, and tumor-specific T-cells were increased [107–110].

Fujita et al., and Veltman et al., reported that the targeting of prostaglandin (PG) E2 with cyclooxygenase 2 (COX2) inhibitors demonstrated a significant decrease of MDSCs in mouse models [111, 112]. Recently, the PKR-like endoplasmic reticulum kinase (PERK) pathway is recently drawing attention and the PERK inhibitor showed an improvement of the efficacy of ICI [113]. TOLLIP (toll interacting protein), a signaling adaptor molecule of myeloid cells necessary for the immunosuppressive function of G-MDSCs, is another target. The ablation of TOLLIP reduced tumor formation in colon cancer models, and the adoptive transfer of TOLLIP-deficient neutrophils showed decreased tumor growth and increased T-cell responses [114].

## CONCLUSIONS

It has been suggested that MDSCs play essential roles in immunosuppression not only in multiple pathological conditions such as cancer, autoimmune diseases, and diabetes, but in certain physiological settings such as pregnancy and obesity. MDSCs are heterogeneous immature myeloid cells that possess important actions in immune tolerance and tumor expansion. ICI therapies have been developed and demonstrated surprising outcomes in many types of cancer. However, the effects of ICIs are not universal or uniformal in all cancer patients, and emerging evidence has indicated that MDSCs are a crucial target to overcome this important issue with a growing understanding of the roles of MDSCs, variable therapeutic strategies and agents targeting MDSCs are under exploration, some of which have been used in clinical trials. More studies are required for the development of more effective strategies against MDSCs.
